# Predictive validity of parent- and self-rated ADHD symptoms in adolescence on adverse socioeconomic and health outcomes

**DOI:** 10.1007/s00787-017-0957-3

**Published:** 2017-02-10

**Authors:** Ebba Du Rietz, Ralf Kuja-Halkola, Isabell Brikell, Andreas Jangmo, Amir Sariaslan, Paul Lichtenstein, Jonna Kuntsi, Henrik Larsson

**Affiliations:** 1 MRC Social, Genetic and Developmental Psychiatry Centre, Institute of Psychiatry, Psychology and Neuroscience, King’s College London, De Crespigny Park, London, UK; 2 Department of Medical Epidemiology and Biostatistics, Karolinska Institutet, Stockholm, Sweden; 3 Department of Medical Sciences, Örebro University, Örebro, Sweden

**Keywords:** ADHD, Developmental epidemiology, Rating scale, Validity

## Abstract

There is scarcity of research investigating the validity of self-report of attention deficit hyperactivity disorder (ADHD) symptoms compared to other informants, such as parents. This study aimed to compare the predictive associations of ADHD symptoms rated by parents and their children across adolescence on a range of adverse socioeconomic and health outcomes in early adulthood. Parent- and self-rated ADHD symptoms were assessed in 2960 individuals in early (13–14 years) and late adolescence (16–17 years). Logistic regression analyses were used to compare the associations between parent- and self-rated ADHD symptoms at both time points and adverse life outcomes in young adulthood obtained from Swedish national registries. Both parent- and self-ratings of ADHD symptoms were associated with increased risk for adverse outcomes, although associations of parent-ratings were more often statistically significant and were generally stronger (OR = 1.12–1.49, *p* < 0.05) than self-ratings (OR = 1.07–1.17, *p* < 0.05). After controlling for the other informant, parent-ratings of ADHD symptoms in both early and late adolescence significantly predicted academic and occupational failure, criminal convictions and traffic-related injuries, while self-ratings of ADHD symptoms only in late adolescence predicted substance use disorder and academic failure. Our findings suggest that both parent- and self-ratings of ADHD symptoms in adolescence provides valuable information on risk of future adverse socioeconomic and health outcomes, however, self-ratings are not valuable once parent-ratings have been taken into account in predicting most outcomes. Thus, clinicians and researchers should prioritize parent-ratings over self-ratings.

**Electronic supplementary material:**

The online version of this article (doi:10.1007/s00787-017-0957-3) contains supplementary material, which is available to authorized users.

## Introduction

Attention deficit hyperactivity disorder (ADHD) is a neurodevelopmental disorder that can have debilitating effects on individuals throughout the lifespan. Clinical and population-based studies have repeatedly shown that both ADHD diagnoses and elevated ADHD symptoms are associated with increased risk of experiencing serious life outcomes, such as educational and occupational difficulties, traffic injuries, criminal convictions and other psychiatric disorders [1–5]. Developing more effective ways of identifying individuals at risk for these serious life events later in life is important to prevent these adverse outcomes from occurring.

Childhood ADHD has an estimated prevalence of 5.3% (95% CI: 5.0–5.6%) worldwide and often persists into adulthood where the prevalence rate is around 2.5% (95% CI: 2.1–3.1%) [6, 7]. While parents and teachers are used as main sources for establishing diagnoses in children, self-report becomes increasingly important during diagnostic interviews in adolescence and young adulthood in both clinical and research settings.

There is scarcity of research that has studied how well parent- and self-ratings of ADHD symptoms in adolescence predict adverse socioeconomic and health outcomes in adulthood. This is an essential issue to investigate, as prevention of serious outcomes later in life is an important task for clinicians working with ADHD. Results from a study using a clinical adolescent ADHD sample found that low academic achievers, compared to high academic achievers, displayed more ADHD symptoms, although group differences were larger for parent-ratings (medium effect size; Cohen’s *d* = 0.60) than self-ratings (small effect size; Cohen’s *d* = 0.26) [8]. Another clinical study of young adults investigated how strongly parent- and self-reports of ADHD during interviews were associated with life events, including academic, occupational and criminal events, after accounting for reports from the other informant [9]. The study found that parent-reports of ADHD symptoms were significantly associated with all events, while self-reports were only significantly associated to employer-rated ADHD and work performance. The findings suggest that parent-reports of ADHD in young adults are more strongly associated to life outcomes and thus have higher concurrent validity than self-reports of ADHD. Similar results were found in a clinical sample of females with ADHD, where parent-rated ADHD symptoms were significantly associated with a higher number of poor outcomes than self-ratings [10]. A major limitation of these studies is that the majority of outcomes were rated subjectively, by the individuals, their parents or employers, which may have biased the results as associations may become inflated due to common method variance between predictor and outcome. Further, large-scale and longitudinal population-based studies that compare the predictive value of parent- and self-ratings of ADHD symptoms in adolescence are currently lacking.

Prospective studies have shown that ADHD in childhood predicts later adverse life outcomes [5, 11, 12], but less is known about the predictive value of ADHD in adolescence and whether it changes across development. To our knowledge, no study has compared the predictive associations between parent- and self-rated ADHD symptoms across adolescence and life outcomes in adulthood. If one of the two source informants would be superior in predicting serious life outcomes, it would suggest that the source informant provides a more accurate assessment of the individual’s ADHD symptomatology and impairment, as ADHD diagnoses predict a range of adverse life outcomes [1–5]. Prospective studies have for example shown that individuals with ADHD are at increased risk of poor educational and occupational performance [1, 13], traffic injuries [3], substance and violence related crimes and convictions [5, 14] and disorders, such as substance use disorders (SUDs) and suicide attempt [2, 25]. Further, if the predictive strength of ADHD ratings varies across ages, it would be valuable to identify when in development parent- and self-ratings have more or less accurate prognostic values. This in turn could be used to inform clinicians about which informant at which stage in development is more or less valid as a predictor of risk for adverse life outcomes.

In this longitudinal, population-based study, we aimed to examine the predictive associations of parent- and self-ratings of ADHD symptoms in adolescence on assessments of adverse socioeconomic and health outcomes from Swedish national registries. We aimed to (1) compare how well parent- and self-ratings of ADHD symptoms assessed in early and late adolescence predict academic, occupational, social and psychiatric outcomes in young adulthood and (2) examine whether parent- and self-ratings of ADHD symptoms independently predict these outcomes over and above the other informant, to examine whether the source informants provide any unique information. We predicted that parent-ratings of ADHD symptoms would more strongly predict adverse life outcomes than self-ratings based on previous research suggesting that parent-report of ADHD in adolescence has greater construct and concurrent validity.

## Methods

### Sample

This study used data from the Twin Study of Child and Adolescent Development [15]. The target sample consisted of all 1480 twin pairs born in Sweden between May 1985 and December 1986. Data on life outcomes were derived through linkage of several nationwide population-based registers in Sweden, last updated in 2009. Individuals who had either died (*N* = 12; obtained from the Cause of Death Register) or emigrated (*N* = 57; according to the Migration Register) before or during year 2009, when registries were last updated, were excluded from analyses.

Individuals and one of their parents were assessed at two separate time points via mailed questionnaires; at age 13–14 years, 1063 (73%) parents and 2263 (78%) adolescents responded, and at age 16–17 years, 1067 (74%) parents and 2369 (82%) adolescents responded, with a majority of parent-rated information supplied by mothers. Informed consent was appropriately obtained and each wave of data collection was approved separately by the ethics committee of Karolinska Institutet, Stockholm, Sweden.

### Measures

#### ADHD symptoms

Parent-ratings consisted of 11 items from the Attention problem (AP) Scale of the Child Behavior Checklist (CBCL) and self-ratings consisted of nine items from the same AP Scale from the Youth Self-Report form (YSR) [16, 17]. The YSR consists of the same items as those in the CBCL except for ‘nervous movements or twitching’ and ‘stares blankly’. The CBCL and YSR are standardized questionnaires used to rate children’s behavioral and emotional problems exhibited in the past 6 months. The AP Scale, which assesses problems related both to inattention and hyperactivity-impulsivity, has been found to predict DSM (Diagnostic and Statistical Manual of Mental Disorders) diagnoses of ADHD [18, 19] and show good reliability, as well as convergent and discriminative validity [16, 17, 20]. Items were scored on a 3-point scale (0 = not true; 1 = sometimes true; and 2 = often true). The correlation coefficient (*r*) between parent- and self-rated symptoms was 0.36 (*p* < 0.001) at 13–14 years and 0.37 (*p* < 0.001) at 16–17 years of age, which is consistent with previous research [9, 21]. The correlation coefficient for parent-ratings over time was 0.63 (*p* < 0.001) and for self-ratings over time was 0.56 (*p* < 0.001).

#### Outcome information

Data on life outcomes were derived through linkage of nationwide population-based registers in Sweden; unique personal identification numbers enabled accurate linkage [21]. The National Patient Register (NPR) has coverage for psychiatric in-patient care and information on out-patient visits to specialist physicians since 2001, with diagnoses based on the International Classification of Diseases (ICD) [22]. The Prescribed Drug Register includes information on prescribed medical drugs since July 2005. The Migration Register supplies migration dates and the Cause of Death Register includes all mortality dates since 1958. The National Crime Register includes information about all criminal convictions in lower courts since 1973 [23]. The Longitudinal Integration Database for Health Insurance and Social Studies contains yearly assessments of a wide range of socio-demographic factors, including income, marital status, social welfare recipiency, and the highest achieved educational level for all individuals aged 15 years or older since 1990.

##### Education

The academic variable was operationalized as not completing 2 or more years of higher (post-secondary) education, which was coded as a binary outcome (i.e., 1 = not completed, 0 = completed), and was obtained from the Education Register [24].

##### Occupation

The occupational outcome was operationalized as receiving unemployment benefits, which was coded as a binary variable (i.e., 1 = received benefits, 0 = not received benefits), and was obtained from the Longitudinal Integration Database for Health Insurance and Social Studies. Unemployment was indexed by having received any benefits for being unemployed at least once for a period of at least 3 years during or after the age of 20 years (year 2006), which is the age at which you can claim unemployment benefits.

##### Criminality

Criminality was identified through the National Crime Register. Convictions were obtained for substance-related crimes (i.e., making, transfer, possession, or use of illegal substances) and violent crimes (i.e., homicide, assault, threat or harassment, robbery, or arson). Criminality was indexed by any substance-related or violent criminal conviction during or after the age of 17 and 18 years (year 2003; after the last ADHD assessment was completed) and was coded as a binary variable (i.e., 1 = has been convicted, 0 = has not been convicted).

##### Unintentional injury

Unintentional injuries were defined as any serious transport injuries, identified as emergency hospital visits due to transport-related trauma (codes V01–V99 in the International Classification of Diseases, Tenth Revision) via the NPR, which has previously been associated to ADHD in a large population-based study [3]. The outcome was indexed as any transport-related injury during or after the age of 17 and 18 years (year 2003) and was coded as a binary variable (1 = experienced unintentional injury, 0 = not experienced unintentional injury).

##### Psychiatric outcomes

The psychiatric outcomes we studied were suicide attempt, SUDs and ADHD. We specifically chose to study suicide attempt and SUDs as we aimed to examine serious psychiatric outcomes, capturing both externalizing (SUD) and internalizing (suicide attempt) conditions that have previously been associated with ADHD [2, 25].

Suicide attempt was defined as any record of a suicide attempt from the NPR (ICD-8 and ICD-9 codes E950-E959, E980-E989; ICD-10 codes X60-X84, Y10-Y34) during or after the age of 18 years. Suicide attempt was coded as a binary variable (1 = suicide attempt, 0 = no suicide attempt).

SUD was indexed as a diagnosis of any SUD from the NPR (ICD-8 codes 303-304; ICD-9 codes 303-304, 305A, 305X; ICD-10 codes F10-F19) during or after 18 years. SUD was coded as a binary variable (1 = diagnosis of substance use disorder, 0 = no diagnosis of substance use disorder).

Individuals with a diagnosis of ADHD were identified from the NPR (ICD-9 code 314; ICD-10 code F90) by having at least 1 record of in-patient (between January 1, 1987, and December 31, 2009) or out-patient (from year 2001 onwards) care for ADHD. We also classified individuals treated with ADHD medication between 2005 and 2009 as patients with ADHD. These criteria have previously been validated as indicators of an ADHD diagnosis [26].

As only 22 (0.9%) individuals met criteria for ADHD, we did not include ADHD diagnosis as an outcome variable due to insufficient power. The prevalence of having been diagnosed with ADHD in our sample is much lower than in the general population (5.3%). This can be explained by the lack of data on out-patient diagnoses before year 2001 (participants were 15–16 years) and prescribed drugs before year 2005 (participants were 19–20 years). As ADHD is often first diagnosed in childhood, it is likely we have failed to identify individuals that only received a diagnosis in childhood.

#### Statistical analyses

We ran logistic regression models to examine how well parent- and self-ratings could predict life outcomes in young adulthood. We adjusted the standard errors for the clustered data structure (e.g., individuals being nested within twin pairs) using a cluster-robust sandwich estimator. The models were run separately for each informant source (parent- and self-ratings), age group (13–14 and 16–17) and life outcome. We also ran models where both informant-ratings were fitted as predictors in one model, to examine the unique predictive value of parent- and self-ratings after controlling for the other informant. Odds ratios (ORs) are used to quantify the strength of the associations and are presented along with 95% confidence intervals. Area under the receiver operating characteristic curve (AU-ROC) is used to quantify the discriminative accuracy of parent- and self-ratings of ADHD symptoms on the outcomes. We additionally investigated the discriminative accuracy of models including both parent- and self-ratings of ADHD symptoms.

**Fig. 1:**
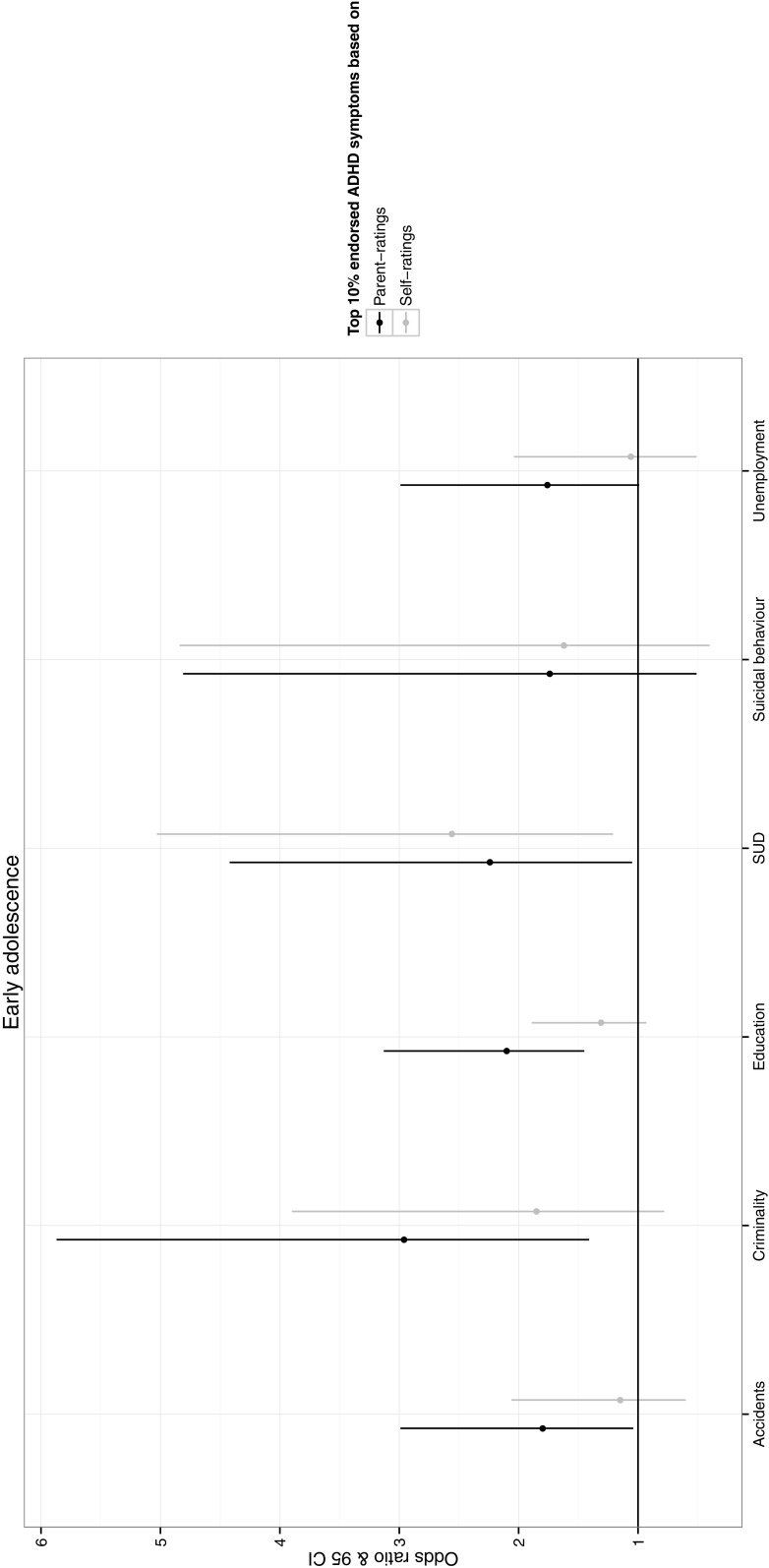
Odds of experiencing each adverse socioeconomic and health outcome if individuals score >90th centile compared to <90th centile on ADHD symptoms rated by each informant in early adolescence. *SUD* substance use disorder

**Fig. 2:**
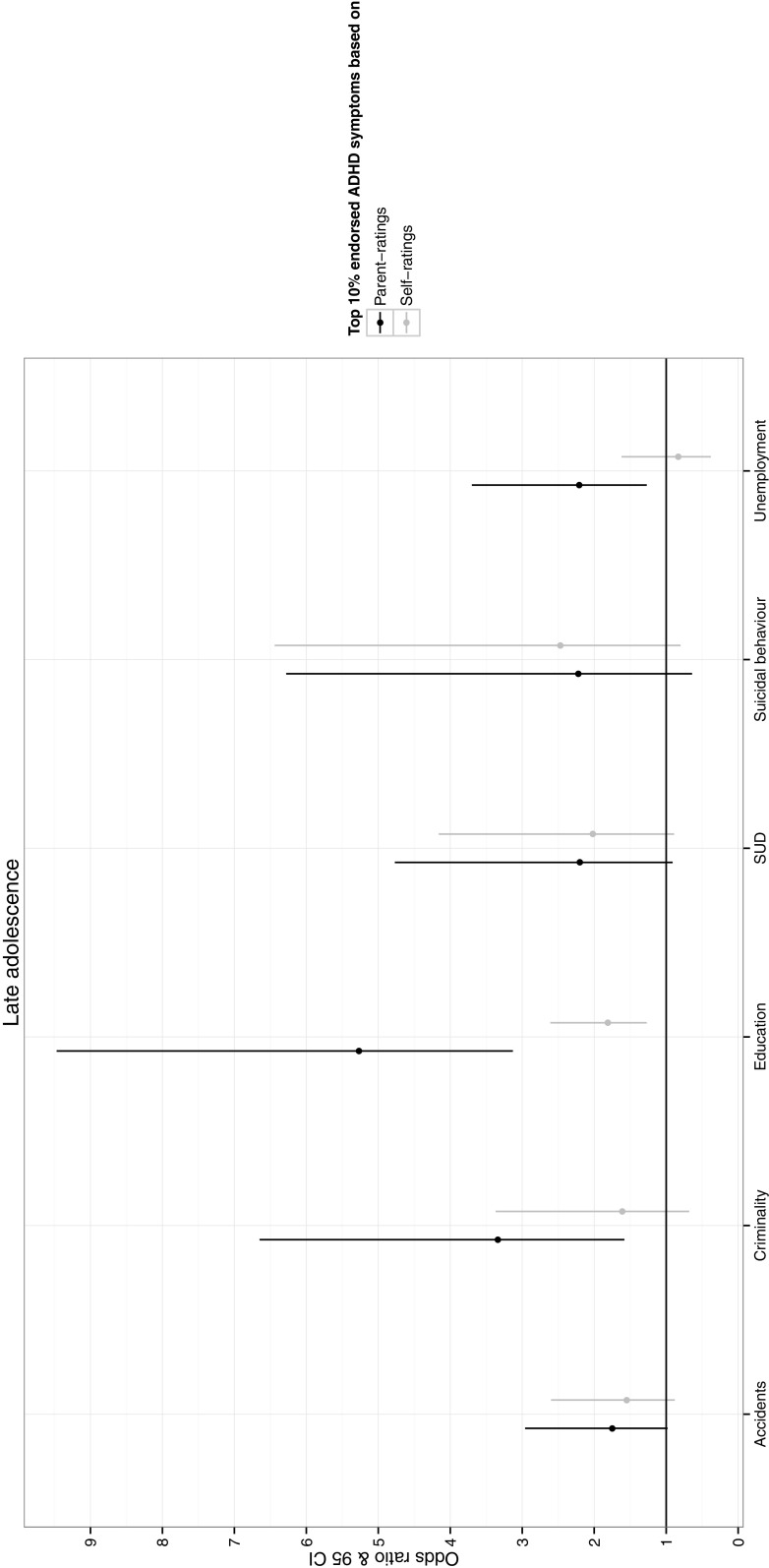
Odds of experiencing each adverse socioeconomic and health outcome if individuals score >90th centile compared to <90th centile on ADHD symptoms rated by each informant in late adolescence. *SUD* substance use disorder

We further dichotomized the ADHD symptom variables using cut-offs at the 90th centile and displayed the odds of experiencing life outcomes if individuals were over or under the 90th centile (Figs. 1, 2). We also ran sensitivity tests using the 95^th^ centile to investigate whether the pattern of results were similar when we used a cut-off which corresponds to the estimated prevalence of ADHD (~5%).

##### Sensitivity analyses

As there were differences in the amount of missing data for self- (*N* = 722 at time 1, *N* = 594 at time 2) and parent-ratings (*N* = 828 at time 1, *N* = 853 at time 2), we ran sensitivity analyses to examine whether differences in data missingness influenced any differences observed in the associations between self- and parent-rated ADHD symptoms and the outcomes. We re-ran the logistic regression models only including cases where neither self- nor parent-ratings were missing. To examine the potential effects of sex, we ran sensitivity analyses on males and females separately.

We additionally re-ran the analyses using parent-ratings of ADHD symptoms while excluding the two items that were absent in the self-rating scale, to examine whether differences in items between the two ratings scales influenced any differences observed in their associations with outcomes. All of the models were fitted in Stata 13 [27].

## Results

See Table 1 for the frequency of adverse socioeconomic and health outcomes and the number of parent- and self-rated ADHD symptoms in early and late adolescence. Adolescents rated their levels of ADHD symptoms as more severe than parents at both 13–14 and 16–17 years.

**Table 1:** Rates of adverse socioeconomic and health outcomes in young adulthood

	Women (*N* = 1507)	Men (*N* = 1436)	Total (%) (*N* = 2944)
Rates (%) of adverse socioeconomic outcomes			
No higher education	1099 (73%)	1163 (81%)	2262 (77%)
Unemployment	71 (5%)	61 (4%)	132 (4%)
Criminal conviction	17 (1%)	80 (6%)	97 (3%)
Rates (%) of adverse health outcomes			
Traffic-related injury	78 (5%)	79 (6%)	157 (5%)
Suicide attempt	24 (2%)	11 (1%)	35 (1%)
SUD diagnosis	38 (3%)	41 (3%)	79 (3%)
Mean (SD) ratings of ADHD symptoms at 13–14 years			
Self-rated	3.86 (2.69)	3.58 (2.71)	3.72 (2.70)
Parent-rated	1.16 (1.86)	1.58 (2.19)	1.36 (2.04)
Mean (SD) ratings of ADHD symptoms at 16–17 years			
Self-rated	4.22 (2.80)	3.38 (2.65)	3.82 (2.76)
Parent-rated	1.12 (1.80)	1.24 (1.96)	1.18 (1.88)

*SUD* substance use disorder,* SD* standard deviation

### Predictive value of parent- and self-rated ADHD symptoms at 13–14 and 16–17 years on life outcomes in early adulthood

#### 13–14 years

Figure 1 depicts the odds of experiencing each life outcome for individuals that score over the 90th centile on the distribution of ADHD symptoms compared to under the 90th centile at 13–14 years. For parent-ratings, the odds of being in an accident, being criminal, not completing higher education, and having a SUD (OR = 1.80–2.96) were significantly higher for individuals in the top 10% compared to the lower 90% of ADHD symptom distribution. For self-ratings, however, only the odds of having a SUD (OR = 2.56) was significantly higher for individuals in the top 10% compared to the lower 90% of the distribution.

The logistic regression analyses showed that parent-rated ADHD symptoms at 13–14 years significantly predicted not completing higher education, unemployment, criminality, unintentional injuries and SUD (OR = 1.12–1.21; Table 2). The discriminative accuracy (AU-ROC) of parent-ratings on these adverse outcomes ranged between 0.58 and 0.65 (Table 3). Self-rated ADHD symptoms at 13–14 years also significantly predicted not completing higher education, criminality and SUD, with slightly lower point estimates (OR = 1.07–1.17) than parent-ratings, but did not predict unintentional injury or unemployment, or any other outcomes (Table 2). The discriminative accuracy (AU-ROC) of self-ratings on the significantly predicted adverse outcomes ranged between 0.55 and 0.62 (Table 3). The discriminative accuracy when both parent- and self-ratings were included in the logistic regression model ranged between 0.58 and 0.65. The AU-ROC values increased slightly when self-ratings were added in the model compared to when only parent-ratings were included for unemployment and SUDs, however, these changes were not significant as the confidence intervals were overlapping (Table 3).

**Table 2:** Predictive value of parent- and self-rated ADHD symptoms across adolescence on adverse socioeconomic and health outcomes in early adulthood

	Parent-ratings OR (95% CI)		Self-ratings OR (95% CI)	
	Crude	Adjusted for self-ratings	Crude	Adjusted for parent-ratings
13–14 years				
No graduate degree	1.21 (1.12, 1.31)**	1.18 (1.09, 1.28)**	1.07 (1.03, 1.11)**	1.03 (0.99, 1.08)
Unemployment	1.13 (1.05, 1.22)**	1.16 (1.07, 1.25)**	1.00 (0.92, 1.08)	0.97 (0.89, 1.05)
Criminality	1.21 (1.11, 1.32)**	1.20 (1.09, 1.32)**	1.11 (1.01, 1.23)*	1.02 (0.91, 1.15)
Injuries	1.12 (1.05, 1.20)**	1.11 (1.02, 1.19)**	1.07 (1.00, 1.14)	1.04 (0.96, 1.12)
Suicide attempts	1.13 (1.00, 1.28)	1.13 (0.97, 1.31)	1.09 (0.95, 1.26)	1.00 (0.85, 1.18)
Substance use disorders	1.15 (1.05, 1.26)**	1.10 (0.98, 1.22)	1.17 (1.06, 1.29)**	1.10 (0.98, 1.23)
16–17 years				
No graduate degree	1.49 (1.35, 1.63)**	1.44 (1.30, 1.60)**	1.15 (1.10, 1.20)**	1.06 (1.01, 1.12)*
Unemployment	1.16 (1.07, 1.25)**	1.16 (1.06, 1.27)**	1.03 (0.96, 1.10)	0.99 (0.92, 1.07)
Criminality	1.29 (1.17, 1.43)**	1.23 (1.06, 1.42)**	1.15 (1.06, 1.25)**	1.09 (0.95, 1.24)
Injuries	1.13 (1.04, 1.22)**	1.11 (1.02, 1.22)*	1.06 (1.00, 1.13)	1.01 (0.93, 1.09)
Suicide attempts	1.19 (1.03, 1.37)*	1.11 (0.93, 1.33)	1.12 (1.00, 1.25)*	1.10 (0.96, 1.26)
Substance use disorders	1.20 (1.07, 1.34)**	1.12 (0.96, 1.30)	1.14 (1.05, 1.25)**	1.13 (1.01, 1.27)*

** *p* value ≤ 0.01, * *p* value ≤ 0.05

**Table 3:** Discriminatory accuracy of parent- and self-rated ADHD symptoms on adverse socioeconomic and health outcomes estimated by the area under the receiver operating characteristic curve (AU-ROC)

	Parent-ratings AU-ROC (95% CI)	Self-ratings AU-ROC (95% CI)	Parent- and self-ratings AU-ROC (95% CI)
13–14 years			
No graduate degree	0.59 (0.56, 0.61)	0.55 (0.52, 0.58)	0.59 (0.56, 0.62)
Unemployment	0.58 (0.52, 0.64)	0.50 (0.44, 0.56)	0.60 (0.54, 0.66)
Criminality	0.65 (0.56, 0.73)	0.59 (0.51, 0.68)	0.65 (0.57, 0.74)
Injuries	0.61 (0.56, 0.66)	0.56 (0.50, 0.61)	0.61 (0.55, 0.67)
Suicide attempts	0.58 (0.47, 0.69)	0.56 (0.44, 0.69)	0.58 (0.46, 0.71)
Substance use disorders	0.59 (0.51, 0.68)	0.62 (0.53, 0.70)	0.60 (0.52, 0.69)
16–17 years			
No graduate degree	0.62 (0.60, 0.64)	0.61 (0.58, 0.63)	0.64 (0.61, 0.66)
Unemployment	0.60 (0.54, 0.65)	0.52 (0.47, 0.58)	0.58 (0.51, 0.65)
Criminality	0.69 (0.61, 0.77)	0.63 (0.56, 0.71)	0.72 (0.64, 0.80)
Injuries	0.57 (0.51, 0.62)	0.55 (0.50, 0.61)	0.56 (0.50, 0.62)
Suicide attempts	0.62 (0.50, 0.73)	0.61 (0.51, 0.72)	0.64 (0.53, 0.75)
Substance use disorders	0.60 (0.50, 0.69)	0.62 (0.54, 0.71)	0.65 (0.56, 0.74)

#### 16–17 years

Figure 2 depicts the odds of experiencing each life outcome for individuals that score over the 90th centile on the distribution of ADHD symptoms compared to under the 90th centile at 16–17 years. For parent-ratings, the odds of being criminal, not completing higher education and being unemployed (OR = 2.21–5.27) were significantly higher for individuals in the top 10% compared to the lower 90% of the ADHD symptom distribution. For self-report, however, only the odds of not completing higher education (OR = 1.81) was significantly higher for individuals in the top 10% of the distribution compared to the lower 90%.

The logistic regression analyses showed that parent-rated ADHD symptoms at 16–17 years significantly predicted all outcomes in early adulthood (OR = 1.13–1.49; Table 2). The discriminative accuracy (AU-ROC) of parent-ratings on the outcomes ranged between 0.57 and 0.69 (Table 3). Self-rated ADHD symptoms significantly predicted all outcomes except for unintentional injuries and unemployment (OR = 1.035–1.15; Table 2). The discriminative accuracy (AU-ROC) of self-ratings on these adverse outcomes ranged between 0.52 and 0.63. The discriminative accuracy when both parent- and self-ratings were included in the logistic regression model ranged between 0.56 and 0.72. The AU-ROC values increased slightly when self-ratings were added in the model compared to when only parent-ratings were included for all outcomes except for unemployment and accidents, however, these changes were not significant as the confidence intervals were overlapping.

### Unique predictive value of parent- and self-rated ADHD symptoms at 13–14 and 16–17 years on life outcomes in early adulthood, over and above the other informant

#### 13–14 years

Parent-rated ADHD symptoms at 13–14 years of age significantly predicted not completing higher education, unemployment, criminality and unintentional injuries when controlling for self-ratings (OR = 1.11–1.20). Self-rated ADHD symptoms did not significantly predict any outcomes in early adulthood when controlling for parent-ratings (Table 2).

#### 16–17 years

Parent-rated ADHD symptoms at 16–17 years significantly predicted not completing higher education, unemployment, criminality and injuries (OR = 1.11–1.44), when controlling for self-ratings. Self-ratings of ADHD symptoms at 16–17 years significantly predicted not completing higher education and SUD (OR = 1.06–1.13) over and above parent-ratings (Table 2).

### Sensitivity analyses

The pattern of results did not change when we excluded cases that had missing values of either parent- or self-ratings (Table S1) or when analyses were run on males and females separately (Table S2 and S3). However, higher self-ratings of ADHD symptoms in late adolescence uniquely predicted less unemployment while controlling for parent-ratings only in males. Further, both parent- and self-rated ADHD symptoms showed slightly higher predictive values on life outcomes in males than in females.

Parent-ratings showed similar associations with the outcomes with and without the two additional ADHD rating scale items, suggesting that differences between parent- and self-rated symptoms in their associations with life outcomes is not explained by differences in rating scale items (Table S4). There was, however, one exception; parent-ratings no longer significantly predicted unemployment over and above self-ratings when parent- and self-rating scales included the same items.

We ran additional sensitivity analyses where we dichotomized the ADHD symptom variables using cut-offs at the 95th centile to explore the odds of experiencing life events for individuals over or under the 95th centile. The overall pattern of results remained the same when we used the 5% cut-off as when the 10% cut-off was used (Table S5).

## Discussion

In this longitudinal, population-based study, we found that both parent- and self-ratings of ADHD symptoms rated in adolescence predicted several important adverse life outcomes in early adulthood. In general, the associations between ADHD symptoms and outcomes were stronger in late compared to early adolescence, and parent-ratings of ADHD symptoms predicted several outcomes over and above self-ratings in both early and late adolescence. The findings suggest that clinicians can rely on both informant sources in adolescence for predicting risk of future adverse socioeconomic and health outcomes, although some caution should be placed on self-ratings of ADHD in early adolescence.

Past research has repeatedly shown that ADHD symptoms rated by parents in childhood are associated with adverse life outcomes, and our current results extend these findings to show that both parent- and self-rated symptoms in adolescence were significantly associated with adverse outcomes. For clinicians, these findings suggest that both informant sources in adolescence may provide valuable information for risk of serious outcomes in adulthood. However, we found that the discriminatory accuracy of both parent- and self-rated ADHD symptoms were low, suggesting that neither can be used to accurately discriminate between adolescents who will experience adverse outcomes from those who will not. Although the response rate of our twin study is high, non-responders are more likely to be male and have higher rates of ADHD symptoms in childhood [28, 29]. Given that previous research indicates that associations between ADHD symptoms and behavioral problems increase along with increasing levels of symptom severity, this may suggest that our observed associations between ADHD symptoms and adverse socioeconomic and health outcomes are somewhat underestimated.

Parent-ratings of ADHD symptoms rated in early and late adolescence were generally more strongly associated with the life outcomes compared to self-ratings, although it should be noted that the confidence intervals of the ORs overlapped for most outcomes, suggesting that the difference between parent- and self-ratings in their predictive strength did not significantly differ. Further, parent-ratings predicted several outcomes over and above self-ratings, and the discriminative accuracy did not significantly improve when self-ratings were added to the models compared to when only parent-ratings were used. Our findings suggest that despite parent- and self-ratings of ADHD symptoms only showing modest correlations (0.36–0.37), self-ratings do not have added value beyond parent-ratings for most life outcomes. While multi-informant approaches (e.g., combining informant source ratings) are commonly used based on the belief that each informant provides unique and valuable information, our results suggests that self-ratings are not valuable once parent-ratings have been taken into account in predicting most outcomes, except for SUDs. One possible explanation for the finding that self-ratings significantly predicted SUDs beyond parent-ratings may be that individuals more likely base their ratings of ADHD symptoms on aspects of behavior related to substance use, which parents may not have as much insight into. Our findings are consistent with other lines of research suggesting that parent-ratings have higher concurrent and construct validity than self-ratings in adolescence [30–32] and young adulthood [10]. For example, one study found higher agreement between parent-ratings of ADHD and clinicians (during interviews with parents) than between self-ratings of ADHD and clinicians (during interviews with children and adolescents) [30]. Another study found that parent-ratings of ADHD symptoms were more predictive of ADHD diagnostic group than self-ratings [31]. Parent-ratings of ADHD have also been found to be more strongly associated with underlying objective (cognitive, neurophysiological and movement) indices of ADHD symptomology than self-ratings [32]. Thus, even though our findings suggest that self-ratings provide some prognostic information, the data indicate that obtaining parent-ratings of ADHD symptoms should be the priority in child and adolescent clinical and research settings.

Both parent- and self-rated ADHD symptoms showed an increase from early to late adolescence in the number of statistically significant associations and also in the effect sizes of the associations with the life outcomes. This overall increase in predictive value is not likely explained by a decrease in endorsed ADHD symptoms with age as parent-rated, but not self-rated, symptoms decreased only slightly (from 1.38 to 1.18). Instead, the findings may reflect the shorter time interval in the predictions with increasing age, or that both parents and adolescents become more accurate in their descriptions of the adolescent’s ADHD symptomatology with age, although this second explanation is unlikely. Future work is needed to extend our findings and examine the predictive validity of parent- and self-rated ADHD in young adulthood. This would be informative in order to explore whether the predictive value of self-ratings increases at a specific point in development from adolescence to adulthood. This could be of great value for both clinicians and researchers when having to decide at which age self-report should be used as the main reporting source of ADHD symptoms.

Sensitivity analyses revealed that differences in results between parent- and self-ratings of ADHD were overall not influenced by (1) differences in missing values or (2) differences in items included in the two rating scales. When we re-ran analyses for sex separately, we found that the pattern of results remained the same, although both parent- and self-rated ADHD symptoms in males showed slightly stronger associations with life outcomes than in females. The stronger associations might reflect that boys tend to display more externalizing features of ADHD than girls [33, 34], which may be more easily captured by rating scales.

It should be acknowledged that this study used a population-based sample, and therefore findings may not generalize to individuals with clinically diagnosed ADHD. Even though behavioral, clinical and etiological research converge in suggesting that ADHD symptoms are the extreme of a normal continuum of behavior in the general population [35, 36], it would be informative to examine the predictive validity of parent- and self-ratings in a clinical sample, especially as individuals in this study rated their levels of ADHD symptoms as more severe than their parents, which is in line with population studies [37] but inconsistent with clinical studies that tend to report higher levels of parent-rated symptoms [31, 32]. Further, the predictive validity of ratings that we have studied may also reflect concurrent validity, as for some outcomes the time period for which the outcomes were indexed in the registers started one year after the 16/17-years assessment wave, and therefore might already have been present during the assessment. Another possible limitation is that the predictive value of ADHD symptoms on life outcomes should be interpreted with caution due to the modest effect sizes for the majority of associations. Further, as the AU-ROC values were low, we conclude that neither parent- nor self-ratings of ADHD symptoms could accurately discriminate between individuals who experienced adverse outcomes to those who did not.

## Conclusion

In conclusion, we found that both parent- and self-ratings of ADHD symptoms in adolescence were significantly associated with adverse socioeconomic and health outcomes in early adulthood. Parent-ratings of ADHD from early to late adolescence were better predictors of serious outcomes, although self-ratings in late adolescence significantly predicted not having a higher educational degree and SUD over and above parent-ratings. Our findings suggest that both informant-ratings of ADHD in adolescence provide valuable prognostic information on risk of future adverse life outcomes. However, clinicians should prioritize parent-ratings over self-rating, especially in younger adolescents.

## Electronic supplementary material

Below is the link to the electronic supplementary material.

Supplementary material 1 (DOCX 30 kb)
