# Supplementary material for: Predictive validity of parent- and self-rated ADHD symptoms in adolescence on adverse socioeconomic and health outcomes
Source: Eur Child Adolesc Psychiatry. 2017 Feb 10;26(7):857–67. doi: 10.1007/s00787-017-0957-3 (PMC5489641; doi:10.1007/s00787-017-0957-3)
Supplement: Supplementary file 1 — Supplementary material 1 (DOCX 30 kb) [file 787_2017_957_MOESM1_ESM.docx]

**Article Title: Predictive validity of parent- and self-rated ADHD symptoms in adolescence on adverse socioeconomic and health outcomes**

**Journal: European Child and Adolescent Psychiatry**

**Ebba Du Rietz^1^, M.Sc., Ralf Kuja-Halkola^2^, Ph.D., Isabell Brikell^2^, M.Sc., Andreas Jangmo^2^, M.Sc., Amir Sariaslan^3^, Ph.D., Paul Lichtenstein^2^, Ph.D., Jonna Kuntsi^1^, Ph.D., Henrik Larsson^2,4^, Ph.D.**

1. King’s College London, UK, MRC Social, Genetic and Developmental Psychiatry Centre, Institute of Psychiatry, Psychology and Neuroscience.

2. Department of Medical Epidemiology and Biostatistics, Karolinska Institutet, Stockholm, Sweden.

3. Department of Psychiatry, Warneford Hospital, University of Oxford, Oxford, UK.

4. Department of Medical Epidemiology and Biostatistics, Karolinska Institutet, Stockholm, Sweden and Department of Medical Sciences, Örebro University, Sweden.

*Corresponding author:*

Ebba Du Rietz

Email: [ebba.du_rietz@kcl.ac.uk](mailto:ebba.du_rietz@kcl.ac.uk)

**Table S1** Predictive value of parent- and self-rated attention-deficit/hyperactivity disorder symptoms across adolescence on academic, occupational and adverse health outcomes; excluding cases with missing values on *either* parent- or self-ratings

|  | Parent-ratings  OR (95% CI) |  | | Self-ratings  OR (95% CI) | |  | |
| --- | --- | --- | --- | --- | --- | --- | --- |
|  | Crude |  | | Crude | |  | |
| ***13-14 years*** |  | |  | |  | |  |
| No graduate degree | 1.20 (1.11, 1.30)** | |  | | 1.07 (1.03, 1.12)** | |  |
| Unemployment | 1.14 (1.06, 1.23)** | |  | | 1.02 (0.94, 1.11) | |  |
| Criminality | 1.21 (1.11, 1.32)** | |  | | 1.10 (0.98, 1.23) | |  |
| Injuries | 1.12 (1.05,1.20)** | |  | | 1.07 (1.00, 1.15)* | |  |
| Suicide attempts | 1.13 (1.00, 1.29) | |  | | 1.05 (0.91, 1.22) | |  |
| Substance use disorders | 1.15 (1.04, 1.26)** | |  | | 1.14 (1.02, 1.26)* | |  |
| ***16-17 years*** |  | |  | |  | |  |
| No graduate degree | 1.49 (1.35, 1.65)** | |  | | 1.13 (1.08, 1.18)** | |  |
| Unemployment | 1.15 (1.06, 1.25)** | |  | | 1.04 (0.96, 1.11)** | |  |
| Criminality | 1.28 (1.15, 1.42)** | |  | | 1.17 (1.06, 1.29)** | |  |
| Injuries | 1.12 (1.03, 1.22)** | |  | | 1.04 (0.97, 1.12) | |  |
| Suicide attempts | 1.17 (1.00, 1.36)* | |  | | 1.14 (1.02, 1.28)* | |  |
| Substance use disorders | 1.19 (1.06, 1.34)** | |  | | 1.17 (1.07, 1.29)** | |  |

** p value ≤.01, * p value ≤.05

**Table S2** Predictive value of parent- and self-rated attention-deficit/hyperactivity disorder symptoms in females across adolescence on academic, occupational and adverse health outcomes (N=1,507, only females)

|  | Parent-ratings  OR (95% CI) | |  | Self-ratings  OR (95% CI) | |
| --- | --- | --- | --- | --- | --- |
|  | Crude | Adjusted for self-ratings |  | Crude | Adjusted for  parent-ratings |
| ***13-14 years*** |  |  |  |  |  |
| No graduate degree | 1.20 (1.07, 1.35)** | 1.16 (1.03, 1.30)* |  | 1.08 (1.02, 1.13)** | 1.05 (0.99, 1.11) |
| Unemployment | 1.19 (1.07, 1.33)** | 1.18 (1.05, 1.33)** |  | 1.08 (0.99, 1.18) | 1.05 (0.95, 1.15) |
| Criminality | 1.29 (1.13, 1.48)** | 1.30 (1.09, 1.55)** |  | 1.11 (0.91, 1.36) | 0.98 (0.75, 1.29) |
| Injuries | 1.11 (1.00, 1.23) | 1.10 (0.98, 1.25) |  | 1.02 (0.93, 1.12) | 1.02 (0.92, 1.14) |
| Suicide attempts | 1.15 (0.99, 1.35) | 1.15 (0.96, 1.39) |  | 1.11 (0.93, 1.31) | 1.01 (0.83, 1.23) |
| Substance use disorders | 1.20 (1.05, 1.36)** | 1.16 (1.01, 1.33)* |  | 1.15 (0.99, 1.33) | 1.06 (0.91, 1.24) |
| ***16-17 years*** |  |  |  |  |  |
| No graduate degree | 1.47 (1.29, 1.67)** | 1.39 (1.22, 1.59)** |  | 1.17 (1.11, 1.24)** | 1.08 (1.01, 1.15)* |
| Unemployment | 1.24 (1.11, 1.38)** | 1.18 (1.05, 1.34)** |  | 1.10 (1.01, 1.20)* | 1.07 (0.97, 1.18) |
| Criminality | 1.42 (1.21, 1.67)** | 1.30 (1.02, 1.67)* |  | 1.25 (1.08, 1.45)** | 1.25 (0.99, 1.58) |
| Injuries | 1.12 (0.99, 1.27) | 1.14 (1.00, 1.29)* |  | 1.03 (0.94, 1.13) | 0.98 (0.88, 1.09) |
| Suicide attempts | 1.16 (0.96, 1.40) | 1.13 (0.90, 1.43) |  | 1.08 (0.96, 1.22) | 1.06 (0.90, 1.25) |
| Substance use disorders | 1.13 (0.91, 1.40) | 1.08 (0.83, 1.40) |  | 1.10 (0.98, 1.24) | 1.10 (0.92, 1.32) |

** p value ≤.01, * p value ≤.05

**Table S3** Predictive value of parent- and self-rated attention-deficit/hyperactivity disorder symptoms in males across adolescence on academic, occupational and adverse health outcomes (N=1,436, only males)

|  | Parent-ratings  OR (95% CI) | |  | Self-ratings  OR (95% CI) | |
| --- | --- | --- | --- | --- | --- |
|  | Crude | Adjusted for self-ratings |  | Crude | Adjusted for  parent-ratings |
| ***13-14 years*** |  |  |  |  |  |
| No graduate degree | 1.20 (1.08, 1.33)** | 1.18 (1.06, 1.31)** |  | 1.07 (1.01, 1.14)* | 1.03 (0.96, 1.10) |
| Unemployment | 1.08 (0.98, 1.20) | 1.17 (1.06, 1.29)** |  | 0.90 (0.79, 1.03) | 0.86 (0.75, 0.99)* |
| Criminality | 1.15 (1.03, 1.29)* | 1.12 (0.99, 1.26) |  | 1.12 (1.01, 1.26)* | 1.06 (0.93, 1.20) |
| Injuries | 1.14 (1.04, 1.24)** | 1.11 (1.01, 1.23)* |  | 1.11 (1.01, 1.22)* | 1.05 (0.94, 1.17) |
| Suicide attempts | 1.16 (0.96, 1.40) | 1.21 (0.98, 1.51) |  | 1.04 (0.81, 1.33) | 0.90 (0.65, 1.23) |
| Substance use disorders | 1.11 (0.97, 1.28) | 1.05 (0.87, 1.25) |  | 1.20 (1.05, 1.37)** | 1.14 (0.95, 1.36) |
| ***16-17 years*** |  |  |  |  |  |
| No graduate degree | 1.50 (1.32, 1.71)** | 1.48 (1.28, 1.70)** |  | 1.17 (1.09, 1.25)** | 1.09 (1.01, 1.17)* |
| Unemployment | 1.07 (0.96, 1.20) | 1.16 (1.00, 1.34)* |  | 0.90 (0.81, 1.01) | 0.86 (0.75, 0.99)* |
| Criminality | 1.24 (1.10, 1.41)** | 1.17 (0.98, 1.39) |  | 1.17 (1.06, 1.29)** | 1.08 (0.93, 1.26) |
| Injuries | 1.13 (1.02, 1.26)* | 1.09 (0.97, 1.24) |  | 1.10 (1.01, 1.21)* | 1.04 (0.93, 1.15) |
| Suicide attempts | 1.33 (1.11, 1.60)** | 1.23 (0.99, 1.53) |  | 1.14 (0.82, 1.56) | 1.07 (0.81, 1.41) |
| Substance use disorders | 1.25 (1.10, 1.43)** | 1.14 (0.95, 1.37) |  | 1.20 (1.06, 1.36)** | 1.17 (1.00, 1.36) |

** p value ≤.01, * p value ≤.05

**Table S4** Predictive value of parent-rated attention-deficit/hyperactivity disorder symptoms, excluding items not present in the self-rating scale, across adolescence on academic, occupational and adverse health outcomes

|  | Parent-ratings  OR (95% CI) | |  |
| --- | --- | --- | --- |
|  | Crude | Adjusted for self-ratings |  |
| ***13-14 years*** |  |  |  |
| No graduate degree | 1.19 (1.10, 1.28)** | 1.18 (1.09, 1.28)** |  |
| Unemployment | 1.13 (1.05, 1.22)** | 1.00 (0.92, 1.08) |  |
| Criminality | 1.18 (1.08, 1.29)** | 1.18 (1.06, 1.31)** |  |
| Injuries | 1.13 (1.06, 1.21)** | 1.10 (1.02, 1.20)* |  |
| Suicide attempts | 1.15 (1.02, 1.30)* | 1.13 (0.96, 1.32) |  |
| Substance use disorders | 1.16 (1.06, 1.27)** | 1.08 (0.96, 1.21) |  |
| ***16-17 years*** |  |  |  |
| No graduate degree | 1.40 (1.28, 1.52)** | 1.38 (1.26, 1.52)** |  |
| Unemployment | 1.16 (1.07, 1.25)** | 1.03 (0.96, 1.10) |  |
| Criminality | 1.27 (1.15, 1.42)** | 1.21 (1.04, 1.41)* |  |
| Injuries | 1.13 (1.04, 1.23)** | 1.11 (1.01, 1.21)* |  |
| Suicide attempts | 1.18 (1.01, 1.36)* | 1.11 (0.92, 1.33) |  |
| Substance use disorders | 1.16 (1.03, 1.32)* | 1.12 (0.97, 1.30) |  |

** p value ≤.01, * p value ≤.05

**Table S5** Odds of experiencing each adverse socioeconomic and health outcome if individuals score >95% centile compared to <95^th^ centile on ADHD symptoms rated by each informant in early and late adolescence

|  | | Parent-ratings  OR (95% CI) |  | | Self-ratings  OR (95% CI) |  | |
| --- | --- | --- | --- | --- | --- | --- | --- |
| ***13-14 years*** |  | | |  |  | |  |
| No graduate degree | 2.60 (1.44, 5.10) | | |  | 1.23 (0.77, 2.02) | |  |
| Unemployment | 2.00 (0.90, 4.00) | | |  | 1.73 (0.75, 3.55) | |  |
| Criminality | 5.15 (2.12, 10.93) | | |  | 2.78 (1.03, 6.36) | |  |
| Injuries | 2.03 (0.95, 3.95) | | |  | 2.41 (1.23, 4.41) | |  |
| Suicide attempts | 2.28 (0.43, 7.72) | | |  | 1.47 (0.17, 6.03) | |  |
| Substance use disorders | 3.29 (1.30, 7.29) | | |  | 1.01 (0.20, 3.20) | |  |
| ***16-17 years*** |  | | |  |  | |  |
| No graduate degree | 13.32 (4.40, 65.85) | | |  | 1.83 (1.11, 3.14) | |  |
| Unemployment | 1.79 (0.77, 3.69) | | |  | 1.23 (0.47, 2.70) | |  |
| Criminality | 3.37 (1.24, 7.87) | | |  | 1.77 (0.54, 4.51) | |  |
| Injuries | 1.64 (0.71, 3.37) | | |  | 1.61 (0.73, 3.18) | |  |
| Suicide attempts | 1.68 (0.19, 7.01) | | |  | 1.45 (0.16, 5.96) | |  |
| Substance use disorders | 3.67 (1.34, 8.36) | | |  | 1.92 (0.59, 4.94) | |  |
